# Can AI substitute the first reader in chest radiograph screening? A retrospective non-inferiority evaluation

**DOI:** 10.1007/s11604-026-01973-z

**Published:** 2026-03-14

**Authors:** Kotaro Yoshida, Atsushi Takamatsu, Rie Tanaka, Tetsuo Matsunaga, Antoine Choppin, Aya Tonouchi, Satoshi Kobayashi, Takeshi Kobayashi

**Affiliations:** 1 Department of Radiology, Fukui Prefectural Hospital, 2-8-1, Yotsui, Fukui, 910‑0846 Japan; 2 Department of Radiology, Kanazawa University Graduate School of Medical Sciences, Kanazawa, Ishikawa 920‑8641 Japan; 3 College of Medical, Pharmaceutical and Health Sciences, Kanazawa University, Kanazawa, Ishikawa 920-0942 Japan; 4 Ishikawa Health Service Association, Kanazawa, Ishikawa 920-0365 Japan; 5 LPIXEL Inc, Chiyodaku, Tokyo 100-0004 Japan; 6 Department of Diagnostic and Interventional Radiology, Ishikawa Prefectural Central Hospital, Kanazawa, Ishikawa 920-8530 Japan

**Keywords:** Chest radiograph, Lung cancer screening, Artificial intelligence, Non-inferiority evaluation

## Abstract

**Purpose:**

To evaluate whether AI can substitute for the first reader in a double-reading workflow for lung-cancer detection on screening chest radiographs.

**Methods:**

A retrospective analysis was conducted in a screening cohort at Ishikawa Health Service Association that included 155,503 participants undergoing 320,329 examinations between January 2018 and September 2020. From examinations initially identified as suspected lung cancer by the conventional double-reading system (*n* = 2,882), prespecified exclusions were applied, yielding 1,847 examinations for detection-performance analysis. AI-based lesion detection was retrospectively performed using three AI models, and the localization accuracy of the AI outputs was evaluated. Detection performance (AI vs. first readers) was compared using McNemar’s test with a non-inferiority margin of − 0.05 (AI deemed non-inferior if the lower bound of the 95% CI exceeded − 0.05) in two settings: (1) all lesions and (2) pulmonary nodule/mass only. The false-positive rate per examination was estimated using 5,784 normal examinations (5,689 participants) performed between January and June 2018 with ≥ 2-year negative follow-up.

**Results:**

For all abnormalities, each AI model met the non-inferiority criterion relative to first readers and showed higher detection rates (AI detection, 62.5–77.3%; first readers, 59.3%). Similar findings were observed when the analysis was limited to nodule/mass only (AI, 64.5–76.5%; first readers, 59.2%). False-positive frequencies per examination were 0.081 (Software A), 0.065 (Software B), and 0.147 (Software C), versus 0.002 for first readers.

**Conclusions:**

In a retrospective screening cohort, three AI models achieved non-inferior, overall higher detection performance compared with first readers for suspected lung cancer on chest radiographs. Despite higher false-positive rates, AI could feasibly assume the first-reader role within a conventional double-reading workflow while maintaining diagnostic quality. Prospective, multi-center studies are warranted to confirm effectiveness, quantify workflow impact, and assess downstream consequences of AI-assisted single reading.

**Supplementary Information:**

The online version contains supplementary material available at 10.1007/s11604-026-01973-z.

## Introduction

Chest radiography is a simple and accessible screening tool for thoracic diseases, requiring minimal medical resources and exposing patients to only low levels of radiation. It has been widely adopted not only in clinical practice but also in population-based screening of asymptomatic individuals for various purposes, most commonly tuberculosis control and, in a limited number of countries, lung cancer screening [1–4]. In Japan, for example, chest radiography is incorporated into routine health examinations mandated by the Industrial Safety and Health Act and is widely used in occupational health across companies and industries [5, 6].

In clinical practice, chest radiographs are interpreted manually by physicians, whereas in screening programs they are typically read by designated readers who receive regular regionally mandated training, and the need for further diagnostic workup is determined. Because multiple anatomical structures are superimposed on a single two-dimensional image, accurate interpretation demands substantial clinical knowledge and expertise. For this reason, images are often read independently by two physicians, a practice commonly referred to as a double-reading system [7]. In Japan’s chest radiograph-based cancer screening, national guidelines explicitly recommend independent double reading by two physicians with at least one an expert reader [5]. Despite these efforts, manual interpretation remains highly dependent on individual physicians’ diagnostic skills, leading to variability in interpretation as well as concerns regarding physician workload.

Numerous studies have investigated the diagnostic performance of artificial intelligence (AI) in interpreting chest radiographs, and several AI-based software programs have already been approved as software as a medical device and implemented in clinical practice across various countries [8–10]. These AI tools have demonstrated high standalone diagnostic performance and have also been reported to be effective as decision support systems for physicians and radiologists [11–13].

In contrast, relatively few studies have addressed the role of AI in the context of population-based cancer screening programs using chest radiography [14]. If a double-reading paradigm that pairs AI with a single physician can achieve diagnostic accuracy comparable to that of the conventional double-reading system involving two physicians, introducing AI could standardize diagnostic performance, reduce both inter-reader and intra-reader variability, and help alleviate physicians’ workload.

The aim of this retrospective study was to evaluate whether AI software can serve as a substitute for the first reader within the conventional double-reading workflow used in chest radiograph-based cancer screening.

## Methods

Ethics approval and consent. This study was approved by the institutional review boards of Kanazawa University and Ishikawa Health Service Association. As this was an exploratory study, no formal sample size calculation was performed; instead, all eligible cases within the predefined study period were retrospectively included. Both written and oral informed consent for the future research use of clinical data and imaging, not limited to a specific study, was obtained from all health-screening participants as part of the routine checkup process. Participants could withdraw their consent at the time of the checkup or at any time thereafter (opt out), in accordance with the screening program’s opt-out procedure.

### Participants

The study cohort consisted of 155,503 participants who underwent a total of 320,329 chest radiographic examinations as part of routine health screening at Ishikawa Health Service Association between January 2018 and September 2020. All examinations were interpreted using a conventional double-reading system, in which two physicians interpreted each image with at least one being an expert reader, in accordance with the Guidelines for Lung Cancer Screening issued by the Japan Lung Cancer Society. Cases with abnormal findings were subsequently referred for further diagnostic evaluation.

In this system, approximately 70% of examinations were interpreted using a two-step manner: the first reading was performed by one of 13 screening physicians at Ishikawa Health Service Association, and the second reading was conducted by a board-certified radiologist or a board-certified pulmonologist. For the remaining approximately 30% of examinations, the radiographs were independently interpreted by both expert readers (a board-certified radiologist and a board-certified pulmonologist). All readers had completed regular case review sessions or chest radiograph interpretation workshops mandated by the Japan Lung Cancer Society’s Guidelines for Lung Cancer Screening. The first readers (*n* = 13) had a median of 10 years (range, 3–33 years) of experience in interpreting chest radiographs, and the second readers (*n* = 2) had 20 and 40 years of experience, respectively. Of 320,329 examinations, prior chest radiographs were available for comparison in 269,033 examinations. When available, readers could review prior images at the time of interpretation.

Participant characteristics (age at examination, sex, smoking history [Brinkman Index]) and the chest radiograph interpretation results (normal vs. abnormal), as determined by the conventional double-reading system, were extracted from the health-screening results database.

### Image acquisition

Chest radiographs were obtained using digital systems (CXDI-401 C, Canon, Japan; or CALNEO HC [DR-ID1200], Fujifilm, Japan). Imaging parameters were standardized as follows: tube voltage 120 kVp; tube current 200 mA with automatic exposure control; grid ratio, 12:1.

### AI software

Three AI models developed by LPIXEL Inc. (Tokyo, Japan) were assessed. Software A was trained specifically for pulmonary nodule detection, with particular emphasis on sensitivity. Software B was derived from Software A and retrained to reduce false-positive detections while preserving nodule sensitivity. Software C was trained to detect lung opacities, including nodules, interstitial changes, consolidations, atelectasis, granular opacities, and cavitary lesions; however, the output is limited to nonspecific bounding boxes without abnormality category labels. For each model, the AI output consisted of lesion-localization bounding boxes automatically superimposed on the chest radiograph to highlight suspicious findings. The bounding boxes were computed from DICOM-format images and exported for retrospective analysis. The AI models were applied without access to prior examinations, whereas human readers could review prior images when available.

### Detection-performance analysis

To evaluate detection performance for suspected lung cancer, a total of 2,882 examinations were extracted from the chest radiograph results database. These examinations had been identified as requiring further diagnostic work-up for suspected lung cancer by the conventional double-reading system. The following exclusion criteria were applied: (1) cases in which both reads were performed by expert readers; (2) cases with extrapulmonary findings (osseous or extrapleural lesions, cardiac or aortic disease, pleural effusion, or pneumothorax); and (3) cases in which lesion annotation was not feasible in the retrospective analysis.

The detection performance of the first readers for suspected lung cancer lesions was obtained from the health-screening results database. In parallel, the diagnostic performance of each AI model was retrospectively analyzed using the same set of examinations. The validity of AI-based lesion detections was assessed using the following procedure. A board-certified radiologist (20 years of experience), blinded to AI outputs, placed bounding boxes around target lesions. An AI output was counted as a true positive if the center of the manual box fell within the AI-generated box; otherwise, it was counted as a false positive. Lesion type was subjectively classified as one of the following: nodule/mass, consolidation, diffuse opacity, or pleural lesion. Lesion size and conspicuity were also evaluated and recorded; conspicuity was graded on a 4-point scale (1 = very subtle, 2 = subtle, 3 = moderately subtle, 4 = well visible) with reference to Jang et al. [15].

Using the expert second reader as the reference standard, detection performance for the first readers and for each AI model was computed, and paired comparisons between first readers and AI models were conducted using McNemar’s test in two settings: (1) all lesions and (2) analyses restricted to pulmonary nodule/mass only. Non-inferiority was prespecified as a margin of − 0.05; an AI model was deemed non-inferior if the lower bound of the 95% confidence interval (CI) for the paired difference exceeded − 0.05. Statistical analyses were performed in R (version 4.2.1; R Foundation for Statistical Computing).

### Assessment of the frequency of false positives

To assess the frequency of false positives, a normal cohort was assembled. From participants who had been judged as having no abnormalities from January through June 2018 and who remained free of lung cancer or other thoracic abnormalities for at least two subsequent years, a total of 5,784 examinations from 5,689 participants were identified. All of these examinations were used to calculate the false-positive rates for the first readers and for each AI model.

## Results

### Study population

**Fig. 1:**
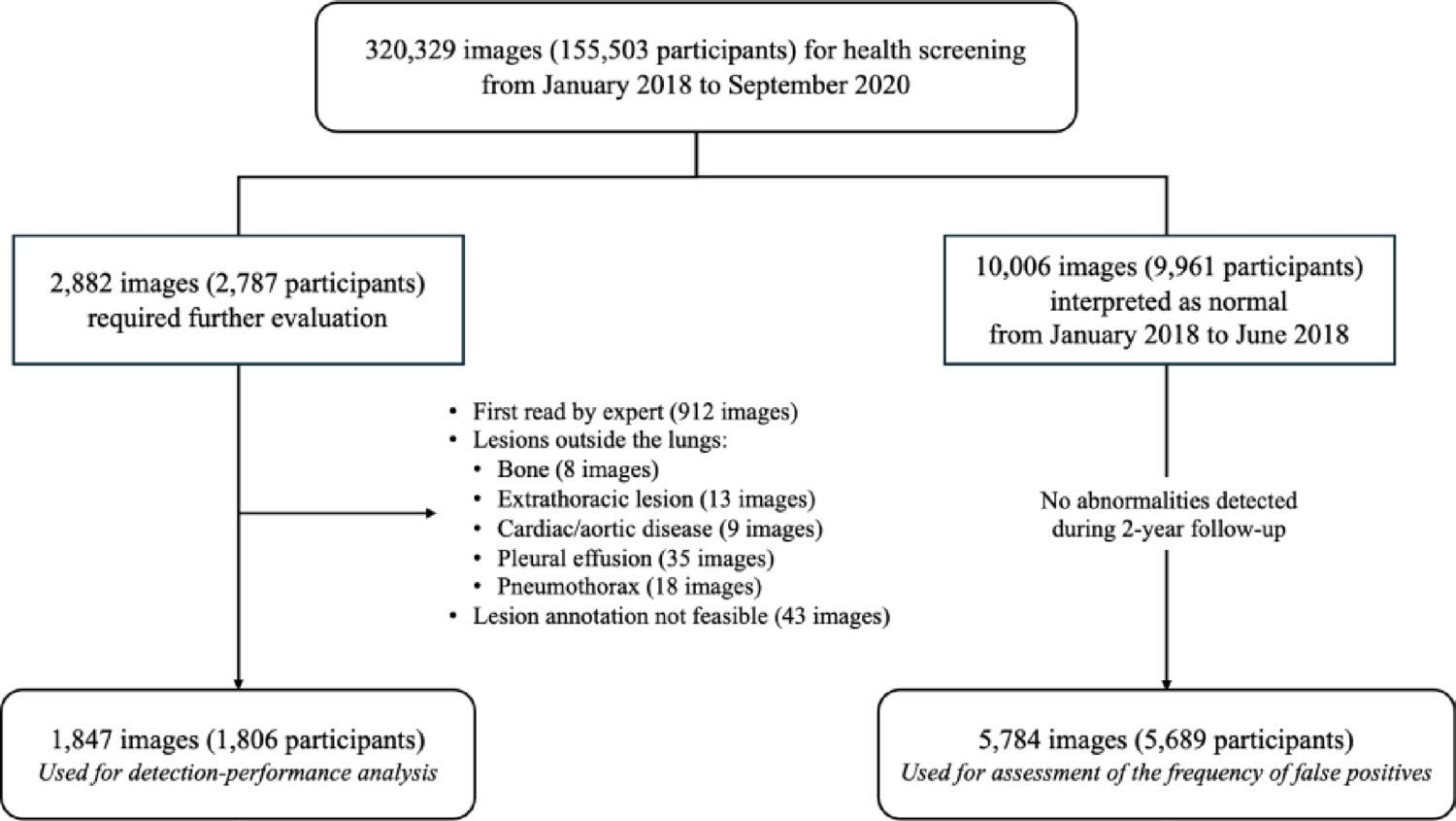
Participant flow diagram for case selection

Figure 1 shows the participant flow through the study. Of the 320,329 images (155,503 participants), 2,882 images were judged as suspected lung cancer based on double reading by two physicians. Of these, 1,035 examinations were excluded according to the predefined exclusion criteria, leaving 1,847 examinations for the final analysis of detection performance. In contrast, among the 10,006 images (9,961 participants) that were judged as normal, 5,784 examinations showed no evidence of lung cancer during 2-year follow-up and were used for the analysis of false-positive frequency.

The clinical characteristics of participants included in the detection-performance and false-positive analyses are summarized in Table 1. Among the 1,847 examinations identified as suspected lung cancer, the mean participant age was 59 years, with a slight predominance of males, and a smoking history was documented in 56% of cases. In contrast, for the 5,784 examinations included for the evaluation of false-positive findings, the mean age was 51 years, also with a slight male predominance, and 59% of participants had a smoking history.

**Table 1:** Baseline characteristics of the study cohorts included in the detection-performance and false-positive analyses

		Detection-performance cohort (*n* = 1,847)	Normal cohort for FP analysis (*n* = 5,784)
Sex			
	Male	1,015 (55)	3,443 (60)
	Female	832 (45)	2,341 (40)
Age at examination, years		59 (40–74)	51 (40–74)
Smoking history (Brinkman Index)			
	Never (0)	816 (44)	2,397 (41)
	Light smoker (1–399)	429 (23)	1,500 (26)
	Heavy smoker (≥ 400)	602 (33)	1,887 (33)

Values are n (%), unless otherwise indicated. Age is presented as median (range). FP = false positive

### Lesion characteristics

The characteristics of the lesions and their conspicuity in the detection-performance analysis are summarized in Table 2. Pulmonary nodules accounted for the majority of findings (*n* = 1,719, 93%), followed by consolidation (*n* = 95, 5%), diffuse opacity (*n* = 24, 1%), and pleural lesions (*n* = 9, 1%).

**Table 2:** Lesion characteristics and visibility scores

		*n* (%)
Lesion type		
	Nodule/mass	1,719 (93)
	Consolidation	95 (5)
	Diffuse opacity	24 (1)
	Pleural lesion	9 (1)
Visibility score		
	1 (very subtle)	168 (9)
	2 (subtle)	620 (34)
	3 (moderately subtle)	545 (30)
	4 (well visible)	514 (28)

Values are n (%). Percentages may not total 100 because of rounding. Visibility was graded on a 4-point scale per Jang et al

### Detection performance analysis

**Fig. 2:**
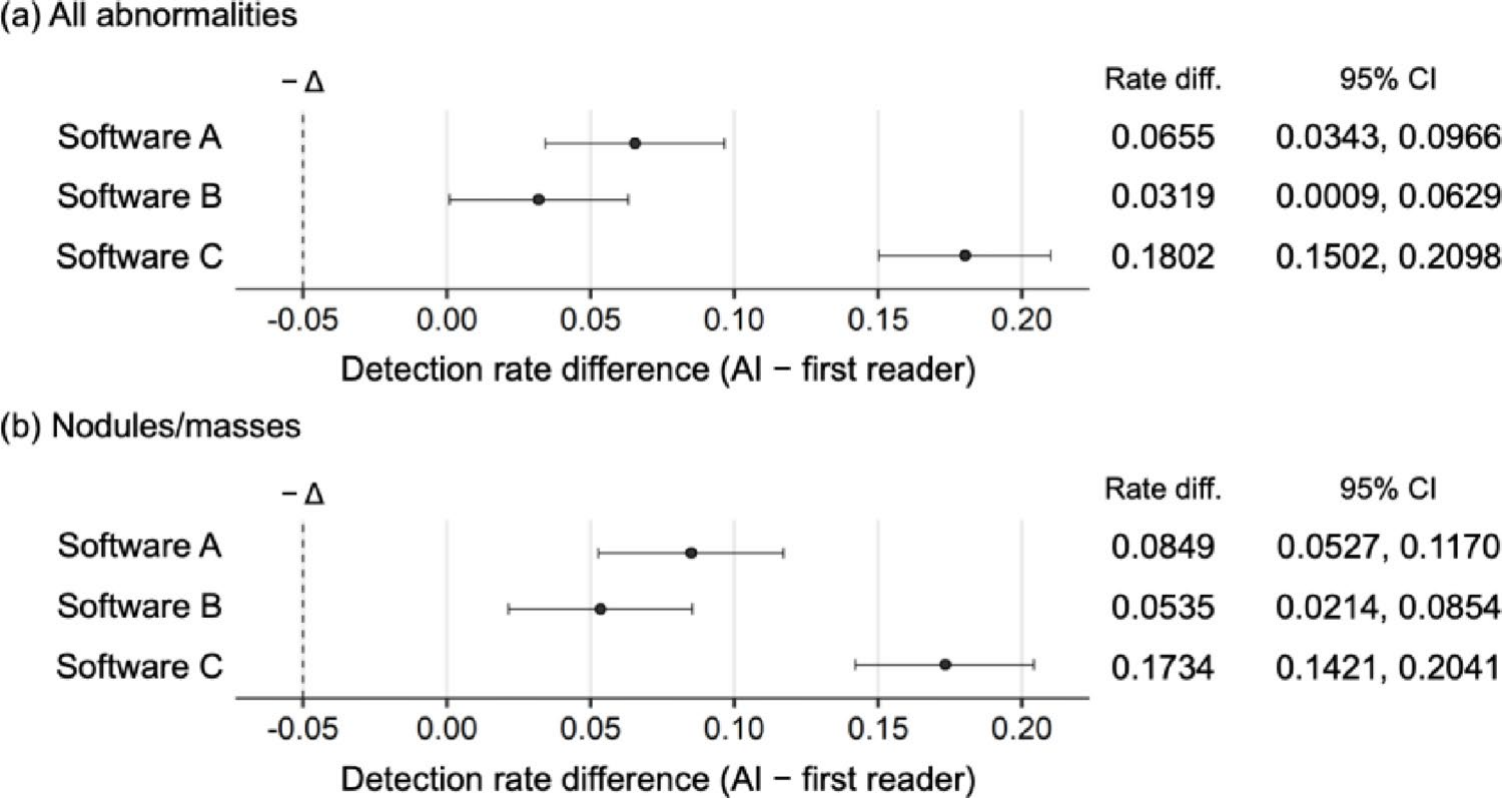
Non-inferiority analysis of detection rate differences between AI models vs. first readers. Forest plots of detection rate differences (AI model minus first reader) with 95% confidence intervals (CIs). The solid vertical line at 0 indicates no difference, and the dashed line at − 0.05 represents the prespecified non-inferiority margin (−Δ). Panels show results for **a** all abnormalities and **b** nodules/masses only. Positive differences indicate higher detection rates for AI models. P values are from McNemar’s test for paired outcomes. Difference = AI − first reader; non-inferior margin = − 0.05; CI = confidence interval

The detection performance of the first readers and each AI model, along with the results of statistical testing, are presented in Table 3; Fig. 2. When all lesions, including non-nodular abnormalities, were analyzed, all three AI models demonstrated non-inferior performance compared with the first readers, with each showing higher detection rates (AI detection, 62.5–77.3%; first readers, 59.3%). Similarly, when the analysis was restricted to pulmonary nodule/mass only, all three AI models again demonstrated non-inferior performance and consistently outperformed the first readers (AI detection, 64.5–76.5%; first readers, 59.2%).

**Table 3:** Detection Performance between first reader and AI models

Cohort	Reader/Model	Detection rate, %
All abnormalities	First reader	59.3
	Software A	65.8
	Software B	62.5
	Software C	77.3
Nodule/mass	First reader	59.2
	Software A	67.7
	Software B	64.5
	Software C	76.5

Values are percentages converted from proportions

Agreement between the first reader and the three AI models is summarized in Supplementary Table S1. Although discordant detections (first reader only vs. AI only) were observed for each AI model, no consistent trend was identified across the three models.

**Fig. 3:**
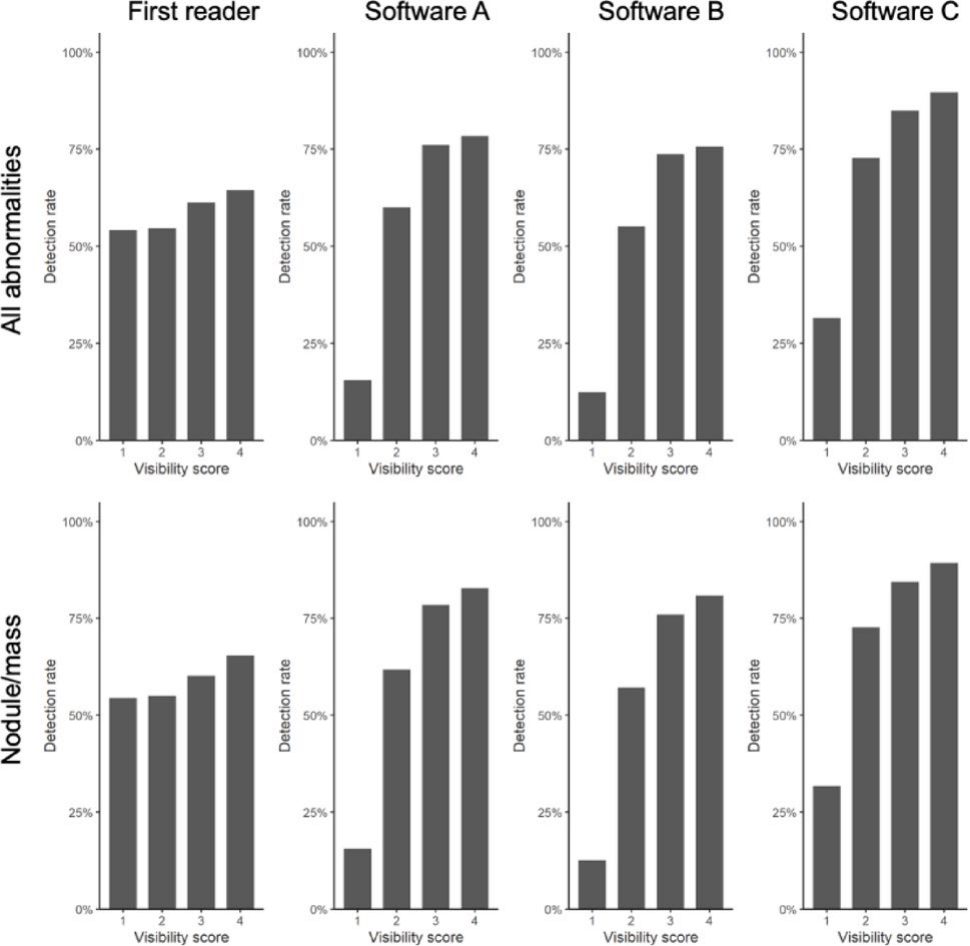
Detection rates stratified by lesion visibility score. Detection rates for first readers and AI models (Software A–C) across visibility categories scored on a 4-point scale (1 = very subtle, 2 = subtle, 3 = moderately subtle, 4 = well visible), referenced to Jang et al. [15]. Detection declined with decreasing visibility, with the sharpest drop observed between scores 2 and 1 on three AI models

**Fig. 4:**
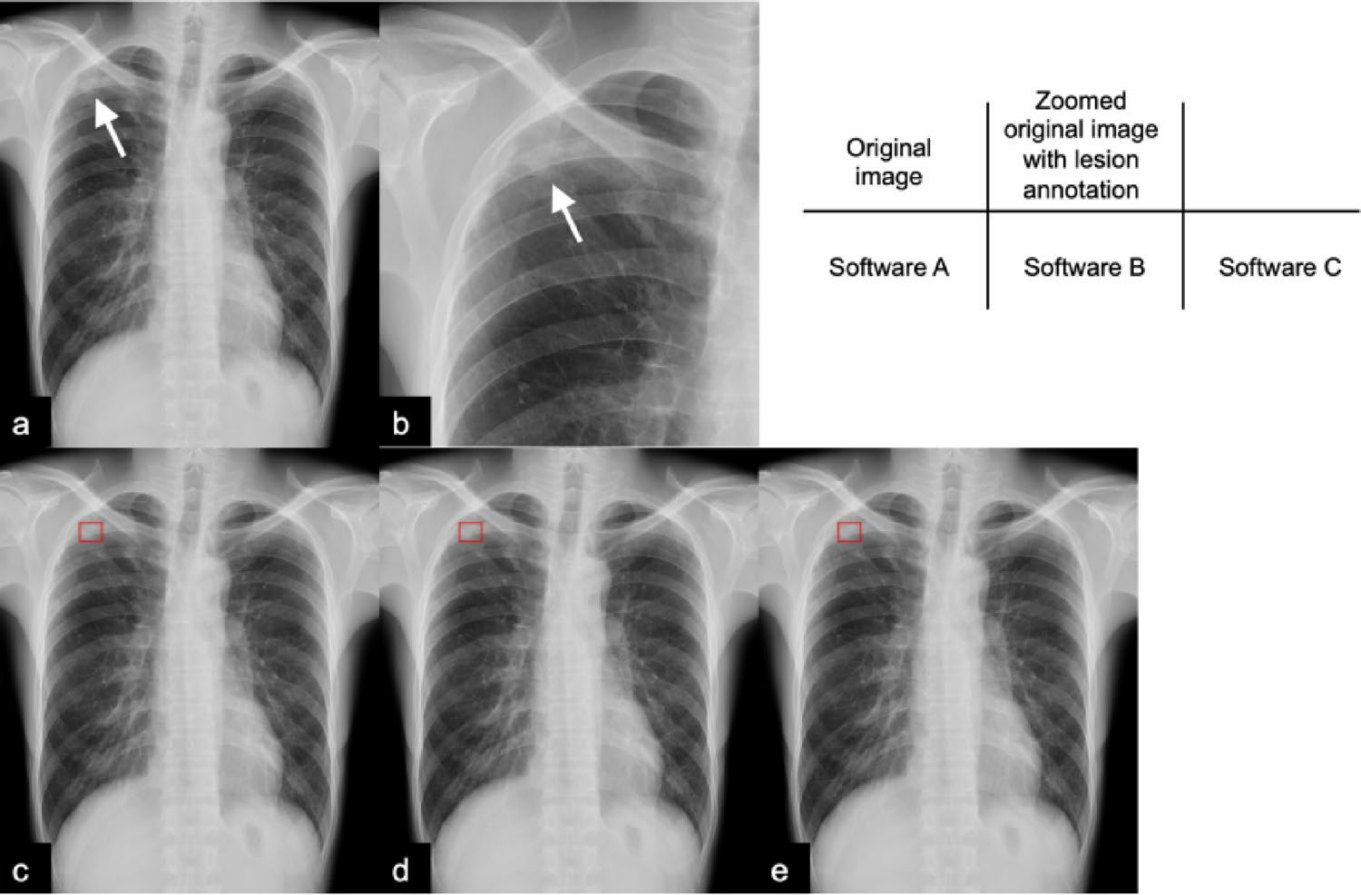
Representative case of concordant detection by first reader and all three AI model (46-year-old, male, BI = 260). ** a** and** b**. Chest radiograph showing a nodule in the right upper lung (arrow). c–e. All three AI models (Software A–C) correctly localized the same lesion, as indicated by red bounding boxes. The lesion’s visibility score was 2 (subtle)

**Fig. 5:**
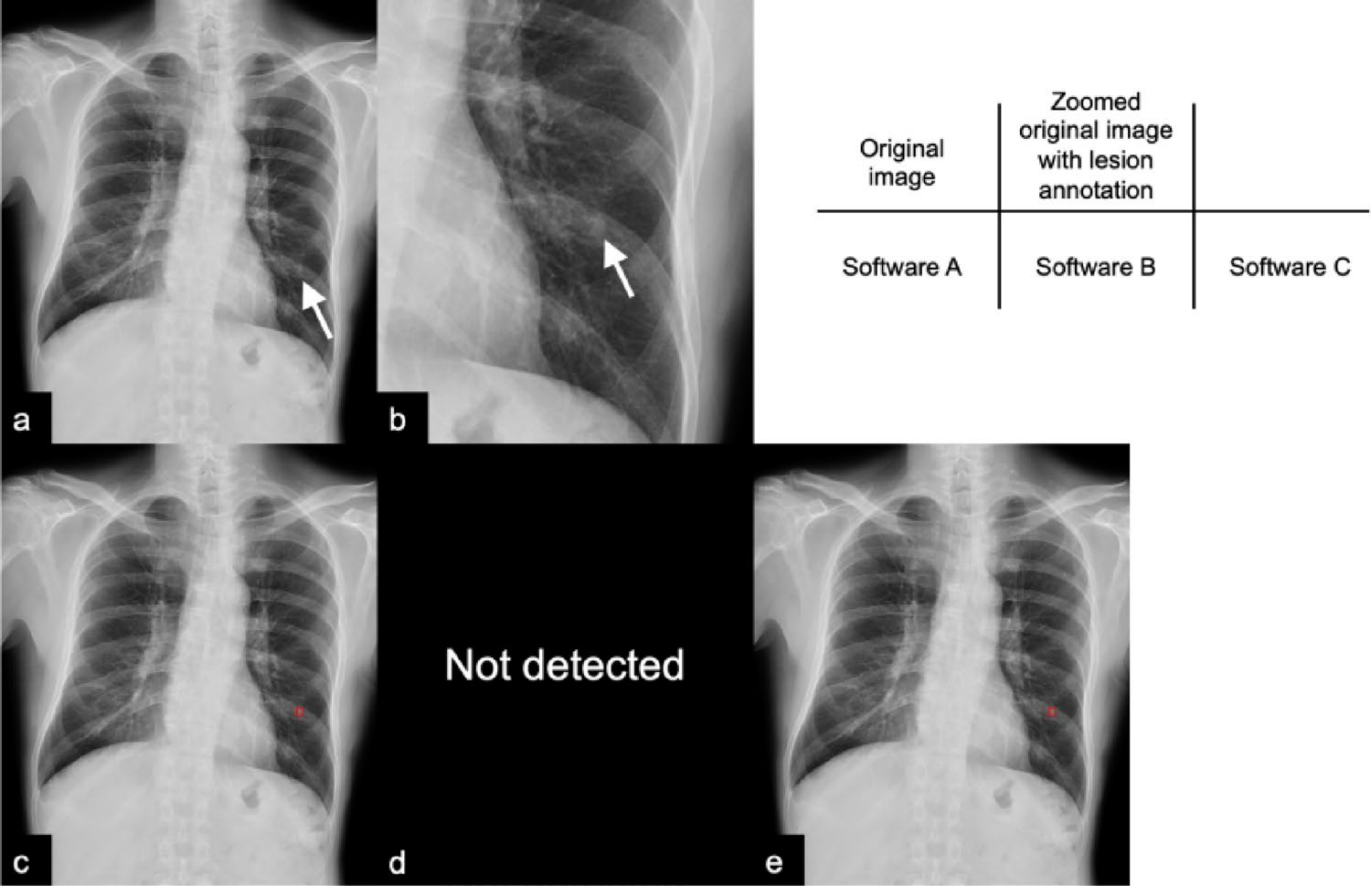
Representative case of the lesion missed by first reader but detected by two AI models (71-year-old, male, BI = 0). **a** and **b **chest radiograph of a lesion in left lower lung (arrow) detected by the second reader. **c**–**e**. The lesion was not detected by the first reader; in the AI outputs, Software A (**c**) and Software C (**e**) correctly localized the lesion (red bounding boxes), whereas Software B (**d**) produced no detection at the target site. The lesion’s visibility score was 2 (subtle)

**Fig. 6:**
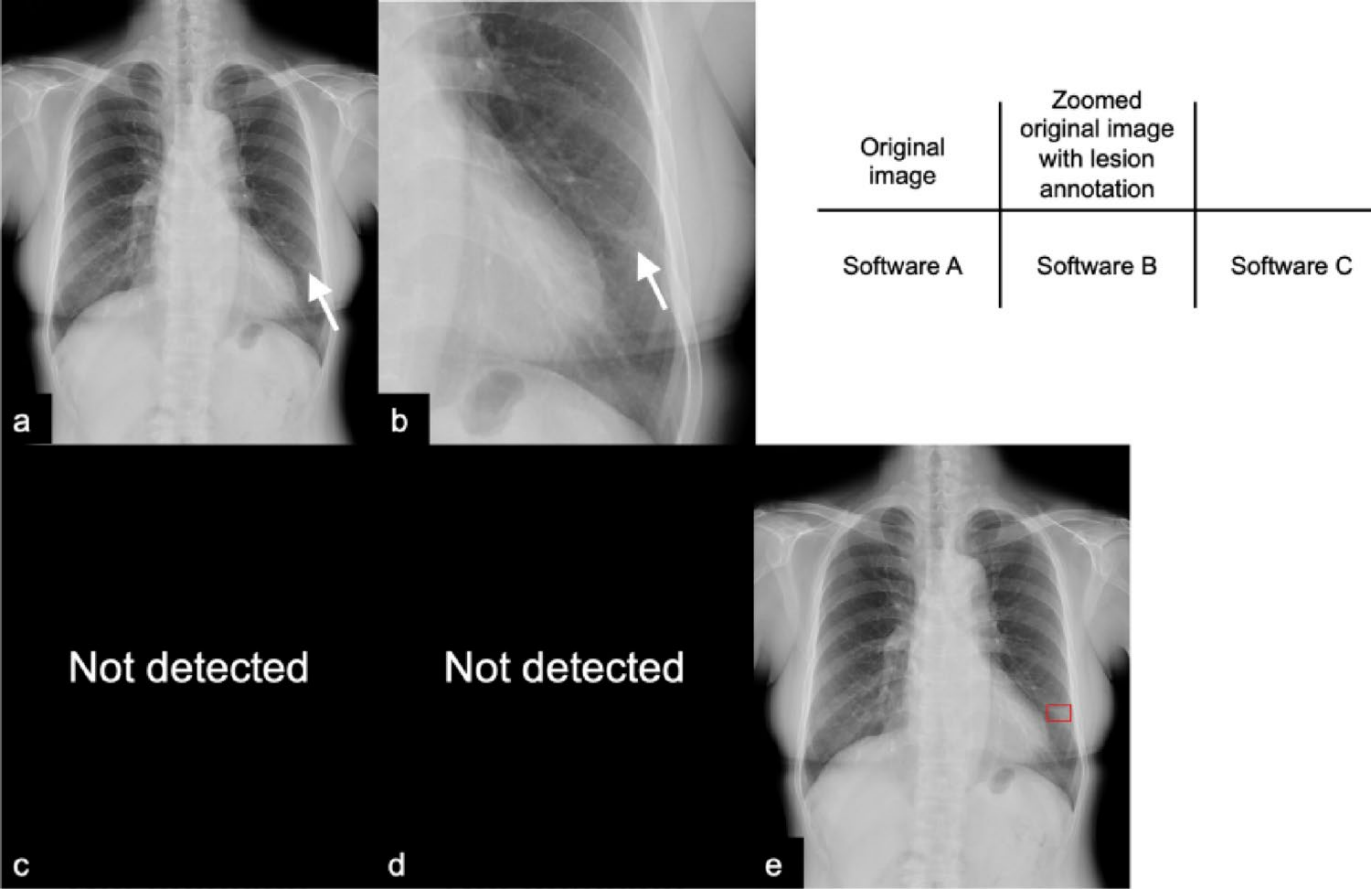
Representative case: lesion missed by first reader but detected by one AI models (66-year-old male, BI = 0). **a** and **b**. Chest radiograph showing a nodule in the left lower lung (arrow) identified by the second reader. **c**–**e** the lesion was not detected by the first reader; in the AI outputs, Software A (**c**) and Software B (**d**) did not detect the lesion at the target site, whereas Software C (**e**) correctly localized the lesion (red bounding boxes). The lesion’s visibility score was 1 (very subtle)

In the subgroup analysis stratified by lesion conspicuity, the detection performance of the AI models decreased for lesions with lower visibility scores, indicating a trend toward reduced sensitivity for subtle nodules (Fig. 3). Three representative cases illustrating detection by the first reader and the AI models are shown in Figs. 4, 5 and 6.

### False-positive analysis

The frequency of false-positive detections per examination was 0.081 for Software A, 0.065 for Software B, and 0.147 for Software C. In contrast, the frequency of false positives in the first readers was 0.002 per examination.

## Discussion

In this retrospective study, we evaluated the diagnostic performance of three AI models for detecting suspected lung cancer on chest radiographs obtained from health screening participants. The main findings were as follows: (1) all three AI models met the predefined non-inferiority criterion relative to first readers in both the overall and nodule/mass-only analyses, and each showed higher detection rates; (2) detection performance declined with decreasing visibility, with the sharpest drop occurring between visibility scores 2 and 1; and (3) false-positive rates differed among the models; Software B had the lowest rate, yet all AI models exceeded human first readers in false positives.

The usefulness of AI software for pulmonary lesion detection has been extensively reported in datasets collected from clinical institutions, with many studies demonstrating diagnostic performance comparable to or even exceeding that of physicians and its utility as a decision-support tool for physicians [12, 13]. However, few have directly addressed its role in population-based cancer screening where the prevalence of disease in this setting is substantially lower than in clinical cohorts. Nam et al. reported in a randomized controlled trial that, for detection of actionable lung nodules by a single board-certified radiologist, AI assistance resulted in higher detection performance than unaided reading [14]. However, to our knowledge, no prior study has specifically examined whether, in a double-reading system for chest radiograph screening of asymptomatic individuals, one of the two human readers can be replaced by AI.

Although competency in interpreting chest radiographs remains a core skill expected of all physicians, the focus of physicians’ education has gradually shifted toward advanced cross-sectional and high-end imaging modalities, and the dedicated time available for training in chest radiograph interpretation has consequently eroded [16]. As a result, maintaining stable, consistently high diagnostic performance in chest radiograph reading over time may become increasingly difficult in routine clinical practice. AI-assisted interpretation is therefore being explored as a practical strategy to sustain accuracy and support human readers in real-world workflows. Our results provide supporting evidence that AI can feasibly replace one physician in the double-reading workflow for lung cancer detection.

In the present study, all three AI models demonstrated non-inferior and, in fact, superior detection performance compared with first readers. Nevertheless, certain nodules remained challenging for AI detection. In particular, small lesions or those with low visibility scores were less likely to be detected by the AI systems in our study. Consistent with prior studies, AI detectability on chest radiographs declines for nodules that are small, subsolid, of low conspicuity, or obscured by overlapping structures [17, 18]. If AI is to assume the role of the first reader in the lung cancer screening workflow by chest radiographs, second human readers will need to be particularly attentive to lesions that are prone to be overlooked by AI.

Another important finding was the relatively higher frequency of false positives with AI compared with first readers—ranging from 0.08 to 0.15 per examination—whereas the rate for first readers was 0.002 per examination. Consistent with prior evaluations, many AI marks proving false-positive arise from non-nodular/benign abnormalities (e.g., scarring/fibrosis) or normal structures, and false positives tend to occur where nodules overlap ribs, clavicles, or hilar vessels, which were not considered suspicious by human readers due to qualitative interpretation [18, 19]. The AI output in this study was limited to lesion candidate detection, and an additional clinical interpretation step to discount findings as benign based on imaging features such as calcification or stability on prior examinations was not incorporated. This difference between detection and clinical interpretation may have contributed to false positive detections. How such AI-generated false-positive findings should be addressed in the second-reading process will be a critical issue for future integration of AI into clinical practice.

This study has several limitations. First, the detection-performance analysis was restricted to examinations that had already been classified as suspected lung cancer by the conventional double-reading system. Therefore, the present results do not represent the overall screening performance across the entire screened population in routine practice. In addition, in real-world clinical settings, readers are required not only to detect lung cancer but also to recognize a broad range of thoracic abnormalities and determine whether further diagnostic evaluation is warranted; this broader interpretive task was not fully captured by our study design. Second, this study was conducted at a single center, so external validity is uncertain and similar results may not be reproduced in cohorts from other institutions. Third, although multiple models were evaluated, they all originated from a single commercial vendor; therefore, the performance of AI systems from other vendors remains unknown. In addition, because the AI models evaluated in this study were research versions that differ from the vendor’s commercially released products, vendor-reported performance and prior reports of the commercial versions cannot be directly compared with our results. Fourth, the assessment of AI non-inferiority was retrospective rather than prospective. Prospective studies should verify that combining AI with a human reader does not raise the referral/recall rate for additional testing. Fifth, the potential time savings associated with AI-assisted second reading were not assessed, and the magnitude of any reduction in reading time is unknown. Sixth, this study was performed within Japan’s unique chest radiograph-based screening system, including mandatory health checkups and a structured double-reading workflow. Accordingly, the generalizability of our findings to screening programs in other countries or healthcare systems may be limited and warrants further validation. Finally, because the false-positive analysis was restricted to examinations with 2-year negative follow-up, human false positives were not defined in this subset; thus, overlap between AI- and human-associated false positives could not be assessed. In addition, the reference standard defined positivity as cases judged to require further diagnostic evaluation based on the secondary read and final determination; we did not systematically verify individual cases through longitudinal follow-up or histopathology.

In conclusion, in a health-screening setting, we compared the lung-cancer detection performance of AI software with that of first readers and found that the AI models were non-inferior. Although AI generated slightly higher false-positive rates, it could feasibly assume the role of the first reader within the conventional double-reading workflow, thereby reducing workload and supporting more consistent care.

## Supplementary Information

Below is the link to the electronic supplementary material.

Supplementary file1 (DOCX 1206 kb)

## Data Availability

Data sharing not applicable to this article as no datasets were generated or analyzed during the current study.
