# Supplementary material for: Can AI substitute the first reader in chest radiograph screening? A retrospective non-inferiority evaluation
Source: Jpn J Radiol. 2026 Mar 14;44(7):1168–76. doi: 10.1007/s11604-026-01973-z (PMC13315103; doi:10.1007/s11604-026-01973-z)
Supplement: Supplementary file 1 — Supplementary file1 (DOCX 1206 kb) [file 11604_2026_1973_MOESM1_ESM.docx]

**Supplementary Table S1. Cross-tabulation of lesion detection: first reader vs. AI software**

|  |  | First reader | |
| --- | --- | --- | --- |
|  |  | Detected | Not detected |
| Software A | Detected | 736 | 480 |
|  | Not detected | 359 | 272 |
|  |  |  |  |
| Software B | Detected | 711 | 443 |
|  | Not detected | 384 | 309 |
|  |  |  |  |
| Software C | Detected | 854 | 574 |
|  | Not detected | 241 | 178 |

Values are the numbers of lesions. “Detected” indicates that the lesion was identified by the first reader or flagged by the AI software, respectively. Each AI program is tabulated separately against the first reader.
